# 

**Published:** 1860-02

**Authors:** 


### Receipts for the Journal up to January 27th, 1860.

| | |
|---|---|
| Dr. L. C. Clark, Ill., Vol 3, | $2 |
| J. W. McKinney, Ill., Vols. 2 & 3, | 2 |
| S. E. Faircloth, Vol. 2, | 2 |
| C. C. Allen, Ill. Vols. 1–2, | 4 |
| J. Williamson, Iowa, Vol. 2, | 2 |
| J. Tripp, Mich., Vols. 1–2, | 4 |
| A. J. Randall, Ill., Vols. 2–3, | 4 |
| Wm. Leachman, Ill., Vol. 2, | 2 |
| C. Goodbrake, Ill., Vol. 3, | 2 |
| James Leedes, Ill., Vol. 2, | 2 |
| J. S. M. C. Van Ness, Iowa, Vol. 2, | 2 |
| B. K. Shurtleff, Ill., Vol. 3, | 2 |
| S. C. Blake, Ill., Vol. 2, | 2 |
| L. N. Dimmock, Ill., Vol. 3, | 2 |
| O. H. Tyler, Mich., Vol. 1, | 2 |
| E. S. Cooper, Cal., Vols. 1–2–½ of 3, | 5 |
| J. H. Griffith, Iowa, Vols. 2–3, | 4 |
| S. M. Alcorn, Ind., Vol. 3. | 2 |
| E. E. Webster, Ill., Vol. 2, | 2 |
| H. Lockwood, Mich., Vols. 1–2, | 4 |
| Chas. Sheperd, Mich., Vol. 3, | 2 |
| Geo Ryan, Ill., Vol. 2, | 2 |
| A. Heavenridge, Ind., Vol. 3, | 2 |
| L. A. Engel, Ill., Vol. 3, | 2 |
| D. O. B. Adams, Ill., Vol. 2, | 2 |
| A. O. Miller, Ill, Vol. 2, | 2 |
| J. C. Smiley, Ill., Vol. 3, | 2 |
| Cloud & Cadwell, Ill., Vol. 3, | 2 |
| H. Steel, Ill., Vol. 3, | 2 |
| B. J. Sapp, Ill., Vol. 3, | 2 |
| J. R. Sikord, Ill., Vol. 3, | 2 |
| J. Dancer, Ind., Vol. 3, | 2 |
| D. B. Wren, Ohio, Vol.. 3, | 2 |
| G. C. Smyth. Ind., Vol. 3, | 2 |
| H. J. Warmouth, Ill., Vol. 3, | 2 |
| John Rea, Ohio, Vol. 3, | 2 |
| Samuel Kelley, Vol. 2, | 2 |
| Hiram Hall, Ill., Vol. 3, | 2 |
| C. M. Smith, Ill., Vol. 3, | 2 |
| S. E. Robinson, Ill., Vol. 3, | 2 |
| H. C. Luce, Ill., Vol. 3, | 2 |
| M. A. Isaac, Ill., Vol. 3, | 2 |
| J. M. C. Dameron, Ill., Vol. 3, | 2 |
| Jas. Thompson, Ill., Vol. 3, | 2 |
| W. Steel, Wis., Vol. 3, | 2 |
| C. S. Jenks, Ill., Vol. 3, | 2 |
| Daniel Kirkpatrick, Ind., Vol. 3, | 2 |
| A. Shepherd, Ill., Vol. 3, | 2 |
| C. Wheeler, Ill., Vol. 3, | 2 |
| G. W. Richards, Ill., Vol. 3, | 2 |
| Abner B. Kimball, Ind., Vol. 3, | 2 |
| R. M. Elliott, Ill., Vol. 3, | 2 |
| John E Handback, Ill., Vol. 3, | 2 |
| J. L. Hooven, Ill., Vol. 3, | 2 |
| W. V. Wiles, Ind., Vol. 3, | 2 |
| F. Bedford, Ill., Vol. 3, | 2 |
| A. S. Brandt, Wis., Vol. 3, | 2 |
| John Bellington, Ill., Vol 3, | 2 |
| O. S. Jenks, Ill., Vol. 3, | 2 |
| T. W. Stull, Ill., Vol. 3, | 2 |
| R. B. Haydon, Mich., Vol. 3, | 2 |
| J. A. Hatch, Ill., Vol. 3, | 2 |
| Solomon Blood, Wis., Vols. 2–3, | 4 |
| A. Hager, Ill., Vol. 2, | 2 |
| Samuel Kelly, Ill., Vol. 2, | 2 |
| J. S. Collings, Ind., Vol. 2, | 2 |
| A. Berchleman, Ill., Vol. 3, | 2 |
| J. S. Brengle, Ill., Vols. 2–3, | 4 |
| R. J. Stough Ill., Vols. 1–2, | 4 |
| W. R. Stone, Ind., Vols. 2 3, | 4 |
| J. R. Bradway, Cal., Vol. 2–½ of 3, | 3 |
| B. F. Newland, Ind., Vols. 1–2, | 4 |
| S. C. Johnson, Wis., Vol. 2, | 2 |
| J. J. Morgan, Iowa, Vols. 1–2–3, | 6 |
| W. L. May, Ind., Vols. 2–3, | 4 |
| B. Wilson, Ill., Vol. 2, | 2 |
| G. W. Hartman, Ill., Vol. 3, | 2 |
| A. Hager, Ill, Vol. 3, | 2 |
| L. D. Personett, Ind., Vol. 3, | 2 |
| F. Ehrhards, Ill., Vol. 2, | 2 |
| R. W. Hall, Iowa, Vol. 3, | 2 |
| W. Vanneys, Ind., Vol. 2, | 2 |
| John H. Hollister, Ill., Vol. 2, | 2 |
| W. H. Gifford, Ind., Vol. 2, | 2 |
| J. F. Spilman, Ill., Vol. 2, | 2 |
| J. F. Williams, Mich. Vol. 3, | 2 |
| Abraham Buler, Ill., Vol. 2, | 2 |
| Charles Hill, Min., Vol. 1, | 1 |
| J. D. Blanchard, Iowa, Vols. ½ of 1,–2–3 | 5 |
| R. F. Carr, Ill., Vol. 2, | 2 |
| Wm. Manson, K. T., Vol. 3, | 2 |
| S. H. Culver, Ill., Vols. 1–2, | 4 |
| J. R. Jay, Iowa, Vol. 2, | 2 |
| Myron Underwood, Iowa, Vol. 3, | 2 |
| Rowley Morris, Wis., Vol. 3, | 2 |
| L. D. Glazebrook, Ind., Vols. 2–3, | 4 |
| A. C. Douglas, Vol. 3, | 2 |
| James Cady, Wis., Vol. 3, | 2 |

